# 0985. Open lung ventilation improves conditions for right ventricle performance by decreasing pulmonary vascular wave reflections in an experimental model of ARDS

**DOI:** 10.1186/2197-425X-2-S1-P70

**Published:** 2014-09-26

**Authors:** A Santos, E Gomez Peñalver, JB Borges, J Retamal, MI Monge García, G Tusman, A Larsson, G Hedenstierna, F Suarez-Sipmann

**Affiliations:** Hedenstierna Laboratory, Surgical Sciences Department, Uppsala University, Uppsala, Sweden; Intensive Care Medicine, Fundación Jiménez Diaz, Madrid, Spain; Insitituto de Investigación Sanitaria, IIS-FJD, Madrid, Spain; Intensive Care Medicine, Hospital del SAS de Jerez, Jerez de la Frontera, Spain; Anesthesiology, Hospital Pirvado de Comunidad, Mar del Plata, Argentina; Anesthesiology and Critical Care Medicine, Uppsala University Hospital, Uppsala, Sweden

## Introduction

Impaired right ventricle (RV) function is associated with worse outcome in ARDS. Pulmonary artery pressure waveform analysis provides information about phenomena that affect RV performance. In particular, pulmonary vascular wave reflection (WR) is directly related with RV stress. We hypothesised that open lung ventilation (OLV), compared with conventional ARDS-net ventilation (CV), would improve conditions for RV performance in an ARDS model. This hypothesis was tested by measuring pulmonary vascular wave reflection (WR).

## Objectives

To evaluate the effect of two mechanical ventilation (MV) strategies on WR in an experimental model of ARDS.

## Methods

8 anesthetized and muscle relaxed pigs were submitted to a two-hit lung injury model combining repeated lung lavages with injurious MV. After lung injury was induced, animals were randomized (4 pigs in each group) to one of two strategies of MV: OLV, PEEP 2cmH2O above the PEEP corresponding with the maximal dynamic compliance in a decreasing PEEP trial after a recruitment manoeuvre; or CV, PEEP adjusted according to the ARDSnetwork table. In both groups tidal volume was 6ml/kg, respiratory rate to maintain PaCO2 between 55-65 mmHg and FIO2 to maintain PaO2 55-80 mmHg.

Pulmonary artery (PA) flow and pressure waveforms (1000Hz sampling rate) were acquired by a high-fidelity microtip manometer and an instantaneous transonic pulmonary flow probe placed in the main PA by a small lateral thoracotomy. These signals were used to separate the forward and backward components of the pressure waveform^1^ and quantify WR. The following indexes of WR were calculated: Backward wave amplitude (APbw); Reflection index (RI) which is the ratio between the backward wave amplitude and the sum of backward and forward wave amplitude. Evaluation was done before (BL) and after lung injury (ARDS) and after 4 hours of management in the respective MV strategy.

## Results

We did not find any significant changes by induction of ARDS but both APbw (5.28±1.35 vs 10.85±2.16 mmHg, p=0.021) and RI (0.28±0.04 vs 0.39±0.04, p=0.021) were lower in OLV comparing with CV.

## Conclusions

In this experimental ARDS-model OLV decreased WR in the pulmonary vascular system comparing with CV, indicated that OLV could reduce the stress on the RV and improve conditions for RV performance.
